# Untargeted lipidomics using liquid chromatography-ion mobility-mass spectrometry reveals novel triacylglycerides in human milk

**DOI:** 10.1038/s41598-020-66235-y

**Published:** 2020-06-09

**Authors:** Alexandra D. George, Melvin C. L. Gay, Mary E. Wlodek, Robert D. Trengove, Kevin Murray, Donna T. Geddes

**Affiliations:** 1 School of Molecular Sciences, The University of Western Australia, Crawley, Perth, WA 6009 Australia; 2 Department of Physiology, School of Biomedical Sciences, Faculty of Medicine, Dentistry and Health Sciences, The University of Melbourne, Parkville, Victoria, 3010 Australia; 3 Separation Science and Metabolomics Laboratory, Murdoch University, Murdoch, Perth, WA 6150 Australia; 4 School of Population and Global Health, The University of Western Australia, Crawley, Perth, WA 6009 Australia

**Keywords:** Lipidomics, Mass spectrometry

## Abstract

Human milk provides the infant with the essential nutritive and non-nutritive factors required for health, growth and development. The human milk lipidome is complex, but comprises predominantly triacylglycerides. Historically, the fatty acid profile of the entire human milk lipidome has been investigated, and many relationships have been identified between infant health and fatty acids. Most of these fatty acids are, however, delivered to the infant as triacylglycerides. Using liquid chromatography-ion mobility-mass spectrometry, the objective of this study was to characterise the triacylglyceride profile of human milk and elucidate relationships between the triacylglyceride profile and infant outcomes in a cohort of 10 exclusively breastfeeding woman-infant dyads. 205 triacylglycerides were identified, including 98 previously not reported in human milk. The dose of specific triacylglycerides differed in relation to infant health, such as lauric acid containing TAGs, which were delivered in significantly higher dose to healthy infants compared to unwell infants.

## Introduction

The lipids in human milk (HM) are vital to the infant, not only as the main source of energy for infant growth, but in their role in immunological interactions and serving as structural components of the infant neural and retinal systems^1,2^. The HM lipidome is complex, with lipids strategically packaged as milk fat globules (MFG). The MFG encapsulates triacylglycerides (TAGs) inside the core, making up 98–99% of the total HM lipidome, while the surrounding MFG membrane is composed of other more polar lipids such as cholesterol and phospholipids^3^. The total lipids are the most variable macronutrient of HM, with sample concentrations varying anywhere between approximately 2 and 100 g/L, increasing throughout a feed, changing throughout the day, and typically increasing throughout lactation^4^. Lipid variations are also reported between women, with maternal diet influencing the fatty acid (FA) composition of the lipidome, but not changing the total lipid content^5,6^. HM FA analysis has classically been carried out using gas chromatography with flame ionisation detection (GC-FID), leading to a wider understanding of the importance of HM lipids for the infant. Relationships have been identified between docosahexaenoic acid (DHA), arachidonic acid (AA), and infant cognitive function, as well as between palmitic acid and infant infection and health^1,7^. FA research using GC-FID, however, does not account for the lipid species from which the FA came (such as the TAG), nor does it consider the FA location on each lipid structure (such as the Sn-1, -2 or -3 position of the TAG glycerol backbone). The positioning of the FA on the TAG glycerol backbone is particularly important because after ingestion, pancreatic and bile salt stimulated lipases preferentially hydrolyse the FA ester bonds from the Sn-1 and Sn-3 positions, resulting in two free FA and one monoglyceride^8^. The functions of the free FA and monoglycerides are different and incorporation of the different FA within the TAG provides the infant with the optimal mixture of FA and monoglycerides^9^.

The TAG profile in milk of different mammalian species has more recently been investigated using liquid chromatography-mass spectrometry (LC-MS) instrumentation^10^. While these techniques are able to differentiate the carbon numbers and/or formula of these TAG species, they are often limited by the number of TAGs identified and studies typically ignore the FA positioning within the TAG. The use of Liquid Chromatography-Ion Mobility-Mass Spectrometry (LC-IM-MS), however, has the capacity to differentiate isomeric TAGs based on their structural arrangements (using collision cross section) and potentially identify more individual TAGs at once, as has been achieved for other lipid species^11^. Furthermore, tandem mass spectrometry allows identification of the fatty acid Sn-2 positioning on the glycerol backbone by interrogation of the size difference of fragment ion spectral peaks^12^. LC-IM-MS lipidomics has been proven with comprehensive TAG characterisation in brain tissue and plasma, as well as bovine milk, however it has not been applied to the HM research field^13–15^.

Here we report the application of an LC-IM-MS method, and the corresponding analytical workflow, for detailed HM TAG profiling. The aim was to achieve not only higher coverage of TAG species than previously achieved in HM, but also to locate the Sn-2 position FA for a better understanding of the delivery of FA from these TAGs to the infant. The potential of this method is demonstrated by comparing analysis of pre-feed and post-feed samples, and samples from different time points in the day, from a cohort of 10 healthy, exclusively breastfeeding women at 3 months lactation. In this study we aimed to focus on the TAG dose that the infant received by simultaneously measuring individual HM production. Our findings report that LC-IM-MS can provide new information into the lipid profile of HM and the discovery of novel HM TAGs.

## Materials and Methods

### Analytical reagents

All solvents and calibrants were purchased from Merck (Sydney, Australia): Methyl *tert*-butyl ether (HPLC grade), Methanol (HPLC grade), Acetonitrile (Supelco, LC-MS grade), Water (LC-MS grade), 2-propanol (LC-MS grade), Ammonium formate (LC-MS grade), Formic acid, Poly-DL-alanine, Leucine enkephalin (HPLC grade), and tripentadecanoin standard.

### Human milk sampling and storage

**Figure 1:**
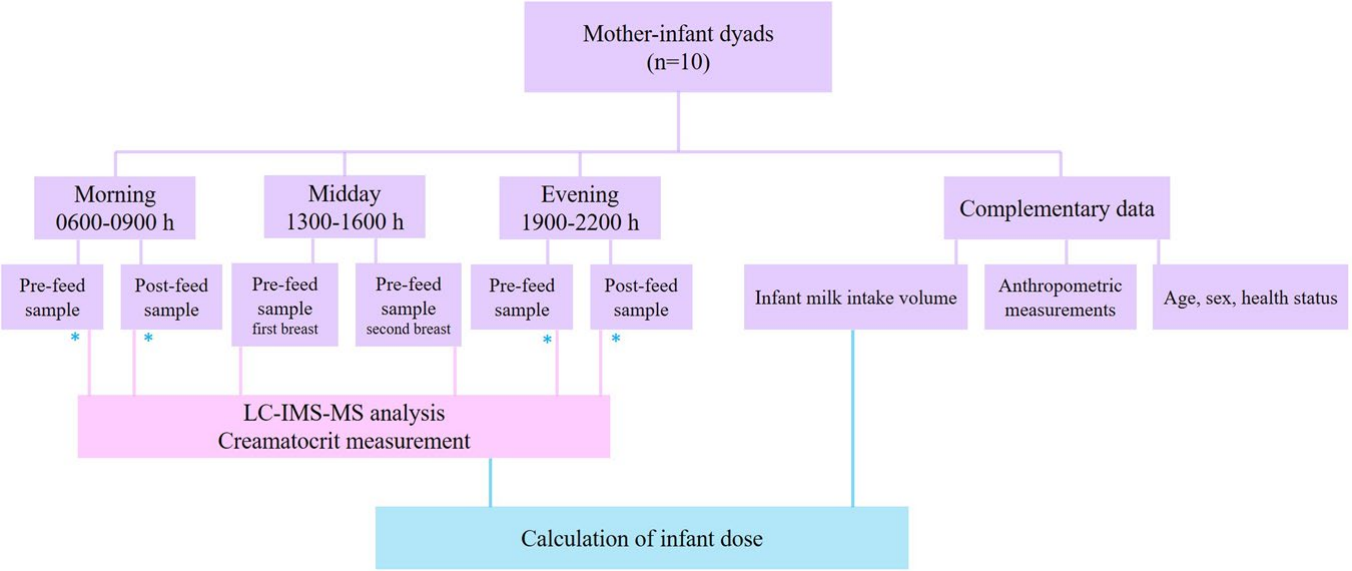
Human milk and complementary data collection workflow at 3 months post-partum, indicating the samples (*) and volume intake used for infant dose calculations.

Human milk samples from 10 healthy lactating women were collected as part of a longitudinal study. Six milk samples of up to 2 mL were manually expressed by mothers into sterile vials at 3 months post-partum (Fig. 1). Pre- (i.e. immediately before feeding) and post- (i.e. immediately after feeding) feed samples were collected between the following times: 0600 and 0900 h, 1900 and 2200 h. Additional pre-feed samples from first and second breast of the feed were collected between 1300 and 1600 h. Samples were stored immediately at −20 °C for less than 48 hours before being transferred to the laboratory freezer for storage at −80 °C prior to analysis. Each sample underwent 1 freeze-thaw cycle. Informed written consent was obtained from all participants for this study. All research was performed in accordance with relevant guidelines and regulations. This study was approved by The University of Western Australia Human Ethics Research Office, RA/4/20/4023.

### Complementary analysis and data collection

Infant and maternal anthropometric measurements, maternal milk production, and other background details such as maternal age and parity, and infant sex were collected. Health status was also reported; infant and mother were listed as ‘unwell’ if cold-like symptoms were reported at time of data collection, or ‘healthy’ if no cold-like symptoms were reported. Infant weight, length and head circumference were measured with Medela Electronic Baby Weigh Scales (Medela Inc., McHenry, IL, USA), Seca 416 Infantometer (Seca, Chino, CA, USA), and a tape measure, respectively. Maternal weight was measured using Seca electronic scales (Seca, Chino, CA, USA). Maternal height was self-reported then confirmed by measuring tape against a wall. Infant milk intake was assessed by measuring the maternal 24-hour milk production, with infant weights taken before and after every feed at 3 months post-partum^16^.

### Lipid extraction

Lipids were extracted from 100 µL HM using a single-phase liquid extraction method consisting of 700 µL *tert*-butyl methyl ether:methanol (50:50 v/v), based on a previously published method^17^. The mixture was vortexed for 1 min followed by 20 min centrifugation at 4000 × *g*. 150 µL of the supernatant, containing extracted lipids, was removed and transferred into a liquid chromatography vial.

### Liquid chromatography-ion mobility spectroscopy-mass spectrometry (LC-IM-MS)

Lipidome profiling of HM samples was carried out using LC-IM-MS instrumentation (ACQUITY UPLC with a SYNAPT G2-S IM-Quadrupole Time of Flight (QTOF) Mass Spectrometer (Waters Corporation, Milford, MA, USA)), optimised based on a previously published method^14^. The autosampler tray was set to 15 °C. Lipid extracts (2 µL injection volume) were separated by an HSS-T3 column (Waters 100 ×2.1 mm, 1.8 µm, 100 Å), kept at 45 °C. A binary solvent system of mobile phase A (acetonitrile:water (60:40, v/v)) and mobile phase B (isopropanol:acetonitrile (90:10, v/v)) was used, both containing 10 mM ammonium formate and 0.1% formic acid. Separation was carried out with a flow rate of 400 µL/min, for a total of 22 min, with the following gradient: 0 min, 60% A; 0–2 min, 57% A; 2–2.1 min, 50% A; 2.1–12 min, 46% A; 12–12.1 min, 30% A; 12.1–18 min, 1% A; followed by 4 minutes of re-equilibration to 60% A. Ion mobility spectroscopy was carried out using nitrogen drift gas and was calibrated with poly-DL-alanine infusion. Untargeted QTOF-MS^E^ mass accuracy was calibrated using leucine-enkephalin, every 30 s, and TAGs with mass ranges of *m/z* 100–1200 were acquired in positive electrospray ionisation mode, with a scan rate of 30 scan/s and collision energy ramp of 20 to 55 eV. Mass accuracy was checked using tripentadecanoin standard solution prior to sample analysis. All samples were analysed in a single batch, with pooled sample quality control (QC) at least every 10 samples.

### Post analytics

Lipid identification was performed on raw data from pooled QC samples. Acquired data was exported from MassLynx version 4.1 (Waters Corporation, Milford, MA, USA) to DriftScope version 2.7 (Waters Corporation, Milford, MA, USA) for calibration. Calibrated data was then exported as a csv file, and analysed using Python scripts produced by Blazenovic *et al*.^13^, altered to search only *m/z* of [NH_4_]^+^ adduct ions and CCS for identification. Each result was confirmed in LipidCCS predictor and fragment ions were manually checked against LipidMaps predicted fragments^18,19^. FA positioning on glycerol backbone was tentatively identified by checking the mass spectra fragmentation patterns, whereby the Sn-2 fatty acid was identified from the spectral peak with the lowest intensity^12^. Results were compiled to produce an initial TAG list containing RT, *m/z* and CCS value for each identified TAG. Solvent blanks and pooled QC samples were used for validation. All pooled QC samples were analysed against the initial TAG list, the relative abundance was compared, and TAGs were removed if they were not identified in all QC samples, and/or if the CoV was above 25%, producing our in-house HM TAG list. To assess the TAG profile of the cohort samples, the raw data for each sample was calibrated in Driftscope and the resulting peak list was exported as a CSV file. These were searched against the HM TAG list using in-house R code (RStudio 2018 v3.5.2), with identification based on RT, *m/z*, and CCS (see Supplementary Material). Parameters of ± 1% error for CCS, ±0.02 Da difference for *m/z*, and ±0.15 min difference for RT were used. TAGs below the limit of detection (250 counts) were considered not present. Identified TAGs were manually searched for in publications that include TAG analysis in human milk, to report if they have previously reported.

GraphPad Prism version 8.2.0 (GraphPad Software, Inc.) was used for statistical analysis. Infant daily milk volume was compared between male and female infants using unpaired t-test. Creamatocrit lipid percentages of samples were converted to concentrations using the following equation *Total lipid concentration(grams/litre)* = *(creamatocrit(%)* − 0.59/0.146)^20^. Total lipid concentration was compared through a feed (pre-feed vs. post-feed, for morning and evening feeds), throughout a day (pre-feed morning vs. noon vs. evening), and between breasts (first breast of feed vs. second breast of feed), using paired t-tests. The concentration of each TAG was calculated from the relative TAG abundance and the total lipid concentration. Variation between samples (different women and different time points) was compared using relative standard deviation. Mixed model with fixed factors of sampling time (morning vs. noon vs. evening) and sampling type (pre-feed vs. post-feed) and random effect of mother was used to examine the TAG concentrations. Total daily TAG intake was estimated by daily milk volume and 98% of the average daily lipid concentration (calculated by averaging the morning and evening, pre- and post-feed concentration, feeds). Daily TAG intake was compared between infants using repeated measures 1-way ANOVA (accounting for maternal effect) and unpaired t-tests were used to compare TAG dose and infant sex and infant health. Relationships between daily TAG intake, infant growth and development, and maternal factors, were investigated using linear regression. Outlier assessment was carried out for all analyses using the ROUT method in GraphPad Prism. Statistical significance was considered at p < 0.05, all reported p values are adjusted for multiplicity, using GraphPad Prism false positive adjustment.

## Results

**Figure 2:**
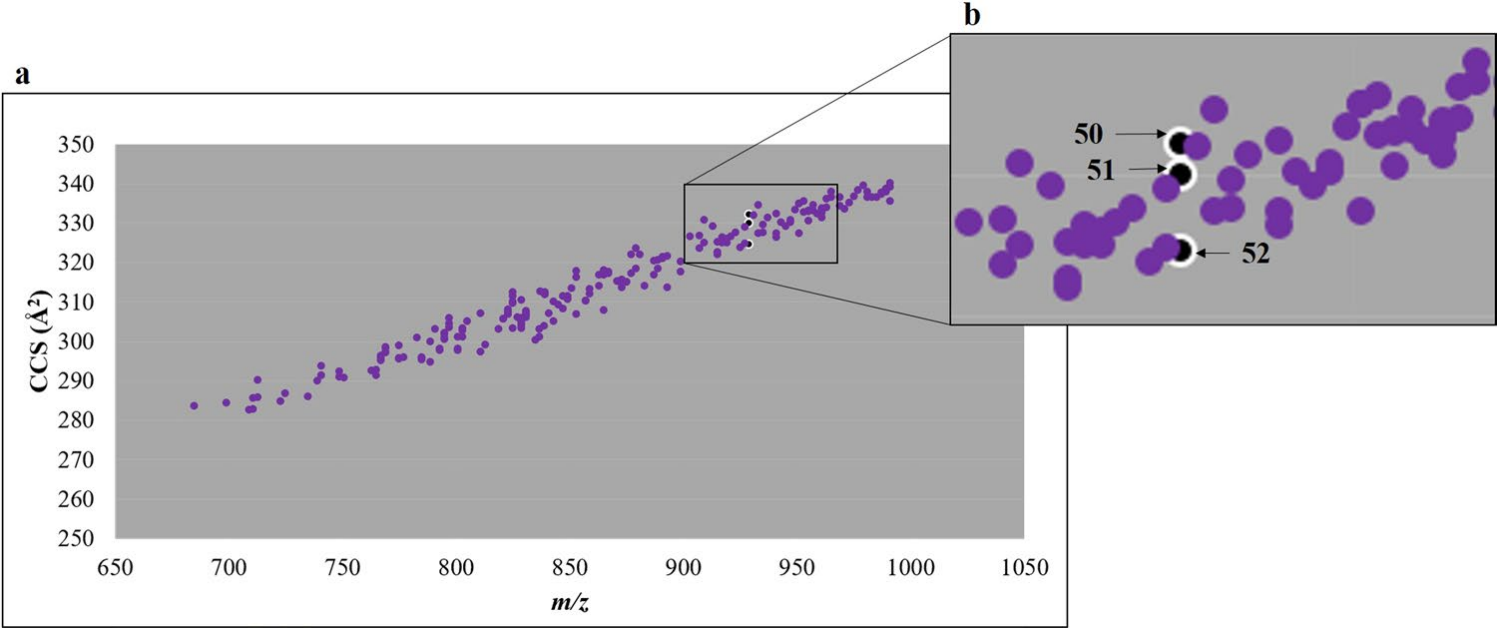
(**a**) Ion mobilogram displaying 205 different TAGs identified in human milk, with *m/z* for each TAG against measured CCS value. (**b**) Separation of C_59_H_106_O_6_ TAGs, TAG50(17:0_/19:0/_20:4), TAG51(18:2_19:1_19:1) and TAG52(18:0_/18:4/_20:0) with m/z 928.8328, by different CCS value.

The LC-IM-MS method identified 205 human milk TAGs (Fig. 2a), from almost 30,000 features. These TAGs were identified based on the precursor and fragment mass (*m/z*), RT and CCS from our in-house HM TAG list (produced from pooled QC samples), and TAGs with the same *m/z* were able to be separated by their CCS values (Fig. 2b).

Supplementary Table 1 describes the characteristics of the 205 HM TAGs. Of the 205 TAGs identified, 98 have, to the best of our knowledge, not been previously identified in HM. In this study the Sn-2 fatty acid was tentatively identified for 120 triacid TAGs using the MS/MS fragmentation patterns, but could not be determined for the remaining 85 diacid TAGs. Supplementary Fig. 1 shows the process of tentatively identifying the Sn-2 position FA, and the overlapping ions within one spectra. No monoacid TAGs were identified.

Ten healthy breastfeeding mothers of term infants provided 6 milk samples for analysis at 3 months post-partum ± 2 days (Table 1). All mothers were exclusively breastfeeding their infant, with one occasionally expressing pumped milk for bottle feeds. Prior to, and following this study, all infants were trending along their respective growth curves and ahead of target with developmental milestones. On the day of collection, 4 infants had cold-like symptoms (referred to as ‘unwell’) and 6 were symptomatically healthy (referred to as ‘healthy’). All mothers were symptomatically healthy.

**Table 1:** Maternal and infant anthropometrics at 3 months post-partum.

Maternal^◊^	Mean ± standard deviation	Range
Age (years)	31.2 ± 2.5	27–34
BMI (kg/m^2^)	26.3 ± 5.6	20.5–39.4
Parity (total offspring)	2.0 ± 1.0	1.0–3.0
**Infant**^◊^		
Weight for age (percentile)	46.8 ± 24.4	11.1–98.8
Weight for length (percentile)	50.4 ± 31.5	6.3–97.5
Length for age (percentile)	47.6 ± 24.3	15.9–89.0
Head circumference for age (percentile)	77.0 ± 21.1	32.4–98.1

◊ n=10 mother-infant dyads.

Daily milk intake for each infant was estimated by 24-hour test weighing resulting in a mean 24-hour intake of 727 ± 137 mL/day (range: 543–894 mL/day) for this cohort. Male infants received 247 mL more milk (p < 0.01) than female infants [95% CI: 177.1, 316.7]. Expressed milk samples (n = 60) were analysed for total lipid content (Table 2). No significant relationships were identified between maternal factors and infant volume intake, or infant anthropometric measurements.

**Table 2:** Concentration of total lipids in human milk samples collected at 3 months post-partum at different time points from 10 study participants.

Sample type^◊^	Mean ± standard deviation (g/L)	Range
Morning, pre-feed	20.9 ± 9.7	8.3–40.5
Morning, post-feed	58.3 ± 32.1	22.5–132.6
Noon, pre-feed, first breast	31.3 ± 17.0	3.5–62.2
Noon, pre-feed, second breast	34.6 ± 15.9	15.1–64.8
Evening, pre-feed	24.2 ± 10.0	12.7–43.9
Evening, post-feed	51.6 ± 18.4	22.6–74.0

◊n=60 samples.

The total lipid concentration increased significantly through both the morning (p < 0.01) [mean difference: 37.35; 95% CI: 19.74, 54.95] and evening (p < 0.01) feeds [mean difference of 27.49; 95% CI: 13.54, 41.44]. The total lipid concentration of the morning feed was not significantly different to that in the evening feed (p = 0.79). The total lipid concentration was significantly different between morning and average noon samples (p = 0.03) [mean difference: 12.05; 95%CI: 3.58, 20.50], but not between breasts (p = 0.64). Mean daily lipid dose to the infant, estimated from lipid concentration and milk intake, was 28.0 ± 12.4 g/day (range: 15.1–56.7). The mean daily lipid intake was higher in healthy infants than in unwell infants (30 g/day compared to 25 g/day), and higher in male infants than female infants (31 g/day compared to 23 g/day), but not significantly different in either case (p = 0.50 and p = 0.30 respectively). No significant relationships were identified between maternal factors and HM lipid concentrations.

### Concentration of TAGs in human milk

**Figure 3:**
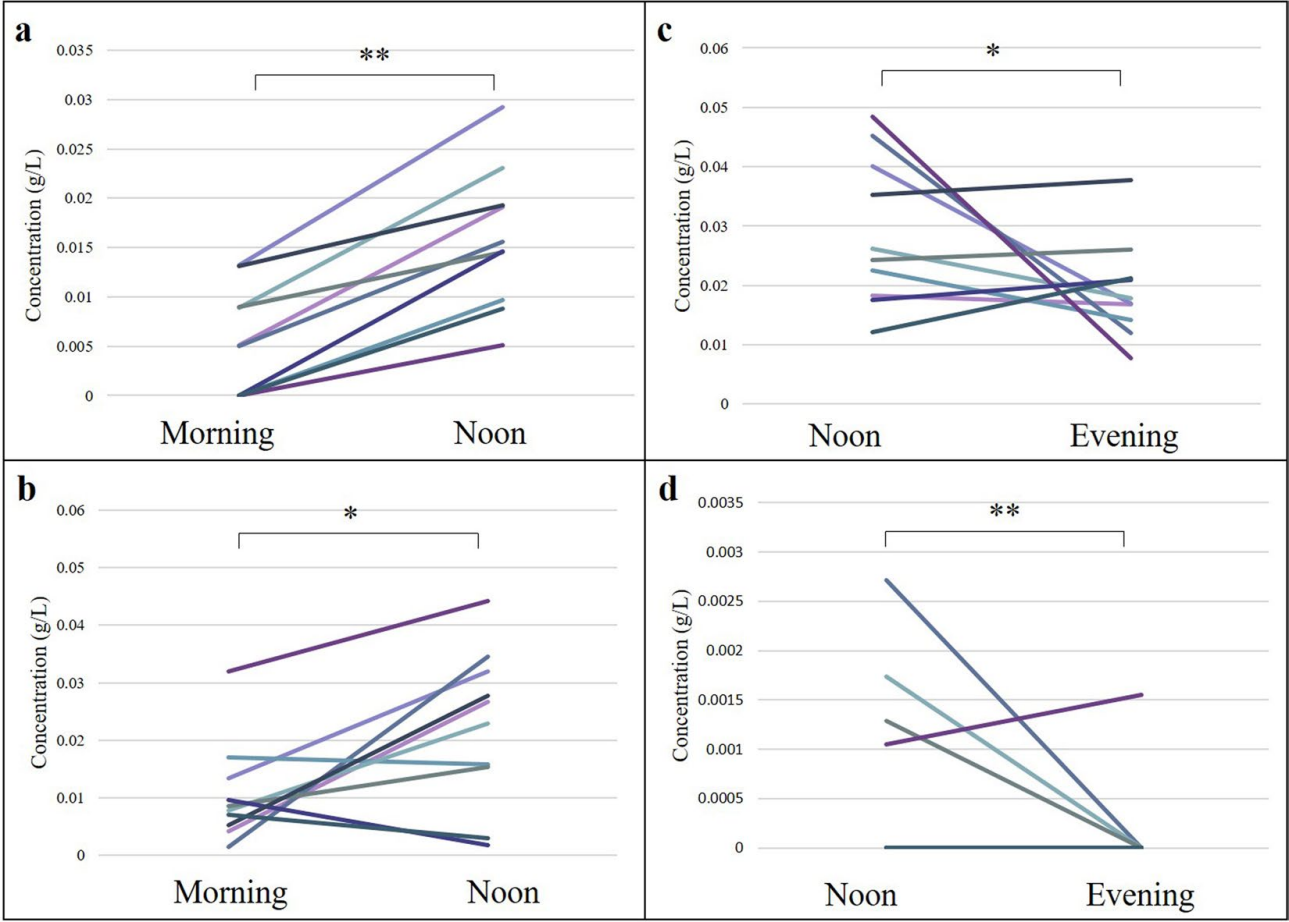
Human milk triacylglyceride concentration changes with significant differences throughout the day (n = 10), (**a**) TAG28(18:1_/20:1/_20:2), (**b**) TAG129(14:0_/20:4/_15:1), (**c**) TAG89(17:1_/16:1/_19:1), (**d**) TAG137(15:0_17:0_17:0).

The concentration of each TAG varied widely between and within women (RSD for all 60 samples for all TAG concentrations ranged from 54.0% and 534.7%). Significant concentration changes occurred in 4 of the 205 TAGs throughout the day (Fig. 3).

There were significant differences between pre- and post-feed samples in both morning (Supplementary Table 2) and evening (Supplementary Table 3) feeds. 22 of the 205 TAGs changed significantly through the morning feed and 25 of the 205 TAGs changed significantly through the evening feed. No differences existed between breasts, nor in comparison of samples from first and second breast. Two specific TAGs did increase significantly between first and second breast, TAG180(12:0_/17:2/_15:1) and TAG199(12:0_14:_14:1) (p=0.01 and p < 0.01 respectively). No relationships were identified between concentration and maternal BMI, age or parity. All sample concentrations and 95% confidence intervals are listed in Supplementary Table 4.

### Dosage of TAGs from human milk

**Figure 4:**
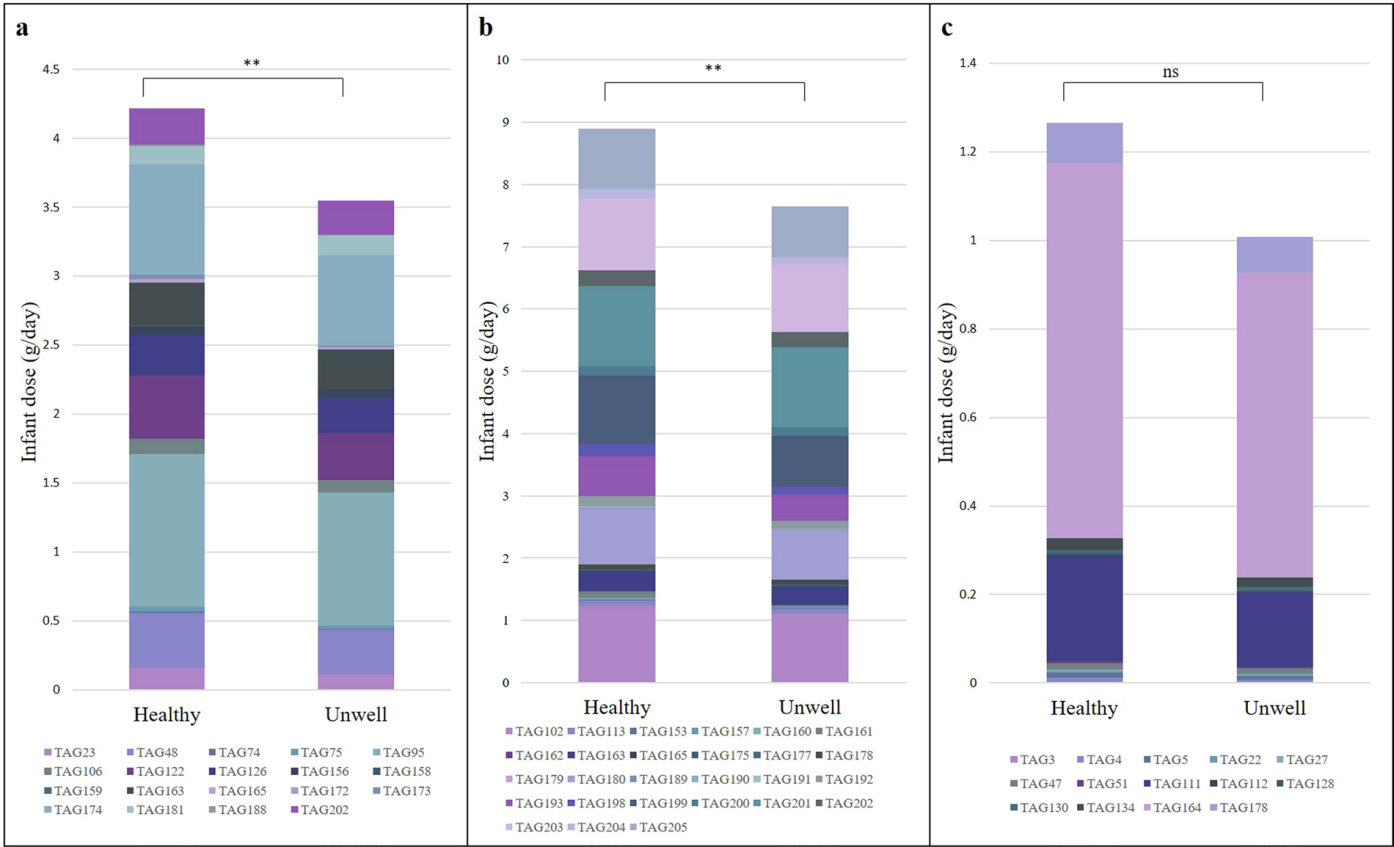
The average daily dose of human milk triacylglycerides containing (**a**) 16:0, (**b**) 12:0 and (**c**) 18:2 fatty acids delivered to healthy and unwell infants (n = 10).

After considering milk intake and estimated TAG concentrations, the infant daily dose for each TAG (g/day) was calculated (Supplementary Table 4). The dose for each TAG varied widely between infants (RSD from 31% to 266%). The total dose of each TAG was assessed in relation to infant growth percentiles (weight for length, height for age and weight for age), using PCA and linear regression, with no identifiable relationships. There was no relationship between infant head circumference percentile and the total dose of DHA- and AA-containing TAGs, both separate, combined and as a ratio. The total dose of eicosapentaenoic acid (20:5), myristic acid (14:0), and oleic acid (18:1)-containing TAGs were not related to infant growth percentile. However, the dose of all palmitic acid-containing (16:0) TAGs and all lauric acid-containing (12:0) TAGs were significantly different between healthy and unwell infants, typically delivered in higher dose to the healthy infant (Fig. 4) (p < 0.01). The dose of linoleic acid-containing (18:2) TAGs was higher in healthy infants than in unwell infants, although not significant (p = 0.15).

The average dose of TAG93(17:0_/13:0/_22:1), TAG193(12:0_12:0_18:3), and TAG199 (12:0_14:1_14:1) were higher in healthy infants and expressed the largest difference compared with unwell infants (0.28, 0.24 and 0.28 g/day higher in healthy infants, respectively), while the average dose of TAG196 (13:0_/15:0/_14:0), TAG181 TAG(14:1_14:1_16:0) and TAG201(12:0_/13:0/_15:1) were higher in unwell infants with the biggest differences (all 0.01 g/day higher in unwell infants), although not statistically significant. No relationships were identified between dose and maternal BMI, age or parity. The infant dose for all TAGs and 95% confidence intervals are listed in Supplementary Table 5.

## Discussion

Human milk lipids are essential to the infant and are involved in not only nutrition and growth, but health and developmental outcomes^21^. In this study we have identified 205 HM TAGs, with 98 not previously published. In contrast to traditional HM FA methyl ester analysis using GC-FID, untargeted lipidomic analysis was carried out with a single phase MTBE lipid extraction, previously validated for HM research and lipidomics, and LC-IM-MS, previously used for bovine milk only, in order to analyse the entire intact TAG profile^13,14,17^.

The added dimension of ion mobility separation to LC-MS increased the number of TAGs identified in a single method to 205, from approximately 80 previously identified at one time (most recently in^22^ and^23^, summarised prior to 2019 in^10^). The CCS derived from IM drift time is unique to each TAG composition and conformation and was able to be used as an additional identifier, proving valuable for resolving co-eluting species even in cases of isomeric TAGs with the same *m/z* (as shown in Fig. 1b). Other studies have also used IM to increase the number of discernible lipid species, in biofluids such as plasma^13,15^. However, unlike in plasma, we identified numerous TAGs containing odd-chain FAs. Human blood was previously thought to have negligible concentrations of these TAGs, as they are not endogenously synthesised, however increasing dairy intake may be the reason for the increased presence of these odd-chain lipids^24^. Serum 15:0, for example, may be indicative of total dairy lipid intake^25^. Similarly, the mammary gland can only synthesize short and medium-chain FA, however the relationship between maternal diet and FA composition in HM has been demonstrated previously^26,27^. Previous FA research in HM has found odd chain FAs, therefore we expected identification of TAGs containing odd-chain FA, such as 17:0 and 19:1, as in TAG13(20:4_/17:0/_22:5) and TAG18(19:1_20:1_20:1)^28^. Maternal dietary sources of fatty acids include foods such as nuts, animal fats, and fish oils, with odd chained FAs primarily derived from consumption of dairy milk and meat (Table 3)^29^. Due to this, future research should include comprehensive dietary data collection.

**Table 3:** Examples of the origin of fatty acids in maternal diet.

Food	Fatty Acids
Vegetable oil	15:1, 16:1, 17:1, 18:1, 18:3
Butter	14:0, 16:0, 17:0, 18:0, 18:2
Salmon	18:4, 20:5, 22:2, 22:5, 22:6
Beef	14:0, 15:0, 20:2, 22:0, 22:4
Tofu	12:0, 14:0, 18:1, 18:2, 18:3
Eggs	16:0, 18:0, 18:2, 20:3, 20:4
Peanuts	16:0, 18:0, 18:2, 20:0, 22:1
Avocado	16:0, 16:1, 18:0, 18:2, 20:1
Bovine milk	12:0, 13:0, 14:1, 17:0, 19:0

Data compiled from Australian Food Composition database^29^

Within the TAG, the FA positioning on the glycerol backbone is critical to its function for the infant. Lipase from the infant gut preferentially digests the Sn-1 and Sn-3 FAs, resulting in an Sn-2 monoglyceride, both of which function differently^30^. It is therefore important to identify and quantitate Sn-2 positioned FAs. In the case of palmitic acid, research suggests that palmitoylglycerol (monoglyceride) is absorbed more readily in the infant gut than the free FA form^31^. Palmitic acid is the major saturated FA of HM and the majority (70%) of it is thought to reside in the Sn-2 position of TAGs^32^. Due to this positional importance we attempted to use the relative abundance of the fragment ion spectral peaks, allocating the smallest peak as the Sn-2 FA. This reasoning is based on the knowledge that the Sn-2 FA fragments least readily, compared to the Sn-1 and Sn-3 FAs on the glycerol backbone, resulting in product ions of lower abundance^12^. Due to the limitation of this method, we were only able to tentatively identify the Sn-2 position of the 120 triacid TAGs. It is not possible to use peak intensities in the case of diacid or monoacid TAGs, due to repeated FAs resulting in larger spectral peaks, even if one was located in the Sn-2 position. Furthermore, the large number of lipids present meant there was overlapping of spectral peaks, potentially affecting interpretation, despite the ability of IM to further separate molecular ions. Due to these issues, our findings could not support the existing research that 16:0 is primarily in the Sn-2 position and this method did not allow elucidation of the Sn-2 FAs with confidence, as is likely in all complex biological fluids^32,33^. Methods capable of better revealing the Sn-2 positioning of TAG FAs, such as collision-induced and ozone-induced dissociation, are promising but are unfortunately not commercially available^34^.

Although we were able to identify TAGs not previously reported, there were some HM TAGs existing in the literature that were not identified. Of particular interest, these include TAGs with FA compositions (14:0/22:6/22:6), (18:1/22:6/22:6) and (18:2/24:0/22:6), and multiple other DHA-containing (22:6) TAGs not identified in this study^35^. In fact, we only identified 8 DHA-containing TAGs in this study. This may be due to the sampling limitation of this small but comprehensive cohort, in which we only compared samples at 3 months post-partum. It is possible that certain TAGs, such as those containing DHA, are found in higher concentrations earlier in lactation in order to facilitate early neural and retinal development^36^. It is most likely that this is a result of diet, and that the women in our cohort ate less fish than those in previously published studies. Overall DHA intake correlates highest with HM DHA content, with fish being the primary dietary origin of DHA FAs^37^. Despite the knowledge that maternal diet may influence HM FAs, lipogenesis in the mammary gland is not well understood. TAG synthesis utilises glycerol and fatty acids obtained via de novo synthesis and from maternal blood, and is thought to be different to that occurring in other organs^38^. This is an area that requires further molecular research in order to enhance our understanding.

Although small, the cohort used in this study was found to be representative of a healthy exclusively breastfeeding population, and consistent with existing knowledge. The range of milk volume productions and therefore infant intake among these women (543–894 mL/day) was above that required for a healthy infant to thrive, and all infants were trending along their respective growth curves appropriately. Daily HM intake was higher in male infants than in female infants, as has previously been published^16^. Total lipid concentrations increased through a feed, as expected, due to both the draining of the breast and the mechanism by which more milk fat globules are released as the feed proceeds^4,39^. Interestingly, the total daily lipid intake did not differ between male and female infants, being offset by a higher volume but lower concentration in males, compared to a higher concentration and lower volume in females^16,40^. HM volume has been indicated as the driving factor for infant growth, but dose may be the main influence for infant development^41^. Regardless of sex, we did find the total daily lipid intake was higher in unwell infants than in healthy infants, a possible response to protect the mother and/or support the infant immune system. The anti-bacterial and anti-viral properties of HM lipids have been reported as likely coming from resulting FA and monoglycerides from TAG digestion^9^. Additionally, HM gangliosides may also be involved in antibacterial functions, by inhibiting bacterial enterotoxins^42^. This difference supports ongoing insight into the responsive nature of human milk, through interaction by retrograde ductal flow of milk during breastfeeding^43–45^. Future analysis of the TAG profile prior to, during, and following infant illness would better indicate the relationship between maternal and infant health, and HM composition.

As with the total lipid content of HM samples, there were significant changes in the concentrations of TAGs through the day, through feeds, and between breasts. Significant changes reported, including an increase in the concentration of more than 20 TAGs throughout a feed, could not be explained by TAG identity (mass or degree of saturation), and are more likely a reflection of the heterogeneity of the breast alveoli and ductal emptying than intentional patterns^46^. Concentration and/or relative abundance of lipids is most commonly investigated in HM lipidomic analysis, however in the case of the impact for the mother-infant dyad, dose is a more appropriate measure to investigate.

The infant daily dose of each TAG was vastly different between infants with up to 266% differences for some TAGs), despite all infants following appropriate growth trajectories and meeting developmental milestones. This was not unexpected as HM is a rich biofluid, specific and unique to the infant, but also influenced by maternal diet^47–49^. In particular, DHA and AA are FAs of great interest in HM research, due to their proposed role in infant cognitive function and visual acuity, yet we did not identify any relationships between DHA- and AA-containing TAGs and infant head circumference or developmental milestones^50^. Although crude, head circumference correlates with brain volume to give the simplest measure of brain development, along with developmental milestone achievements, but with a sample size of 10 healthy infants we are limited in what we can identify^51^. Eicosapentaenoic acid (20:5), myristic acid (14:0) and oleic acid (18:1) have recently been suggested as indicators of infant growth in pre-term infants^52^. These FAs were predicted to be present in higher dose, based on hypothetical volumes (not measured intake), in faster growing infants, yet we saw no relationship between infant growth percentiles (weight and length) and the infant dose of eicosapentaenoic, myristic and oleic acid from TAGs. This may be because our infants were born term, and it is known that preterm infants have different nutritional requirements than term infants^53^. All palmitic acid (16:0), lauric acid (12:0) and linoleic acid (18:2) - containing TAGs were found in higher dose in healthy infants in comparison to unwell infants, suggesting their protective function in HM. Previous studies have indicated relationships between these FAs and health, although one study reported higher total palmitic acid in unwell infants^7^. The suggested roles of TAGs are currently limited to existing knowledge of the FAs that TAGs deliver to the infant, however we did report some differences in TAGs in infants who were healthy compared to those who were unwell. TAG93(17:0_/13:0/_22:1), TAG193(12:0_12:0_18:3), and TAG199(12:0_14:1_14:1) were found in higher dose in healthy infants, potentially contributing to the function of HM in preventing infant illness^53^. In contrast, TAG181(14:1_14:1_16:0), TAG196(13:0_/15:0/_14:0). and TAG201(12:0_/13:0/_15:1) were all found in higher dose in the unwell infants, suggesting a malleable responsive behaviour of HM, by way of retrograde milk flow^43–45^. The likely reason we did not identify previously reported correlations between infant outcomes, or maternal factors, and the TAG profile is likely due to the sample size of this cohort (n = 10). One aspect for future consideration is that the FAs with implications in infant health may not come from TAGs. Due to the presence of many other lipid types it is entirely possible that certain FAs, such as DHA and AA, originate from other such species as sphingomyelin, from which they are incorporated to the infant brain, for example^54^.

In summary, this LC-IM-MS method allowed further investigation of the lipid profile of HM and identified novel lipids not previously published in HM. In particular, many of these novel TAGs contained odd-chained FAs, likely originating from maternal diet. In this small cohort we demonstrated that the dose of some TAGs was different for healthy infants compared to unwell infants. More extensive investigation of these HM TAGs, and other lipid types is indicated, especially in relation to infant dosage and infant outcomes. A deeper understanding of these lipids gives rise to the possibility of guiding maternal nutritional guidelines for optimal infant outcomes.

## Supplementary information

Supplementary Tables.

Supplementary information with Figures.

## Data Availability

The datasets analysed during the current study are available from the corresponding author on reasonable request.
