# Supplementary material for: Untargeted lipidomics using liquid chromatography-ion mobility-mass spectrometry reveals novel triacylglycerides in human milk
Source: Sci Rep. 2020 Jun 9;10:9255. doi: 10.1038/s41598-020-66235-y (PMC7283244; doi:10.1038/s41598-020-66235-y)
Supplement: Supplementary file 2 — Supplementary information with Figures. [file 41598_2020_66235_MOESM2_ESM.docx]

**SUPPLEMENTARY MATERIAL FOR** Untargeted lipidomics using liquid chromatography – ion mobility– mass spectrometry reveals novel triacylglycerides in human milk

**AUTHORS** Alexandra D. George, Melvin C.L. Gay, Mary E. Wlodek, Robert D. Trengove, Kevin Murray, Donna T. Geddes

**Page 1:** Online repository details for untargeted human milk triacylglyceride analysis

Repository can be found here <https://github.com/ADGEORGElipidomics/TAG-search> and contains:

In-house R script

HM TAG list

HM TAG example raw data set

**Page 2:** Supplementary Figure 1A

**Page 3:** Supplementary Figure 1B

**Supplementary tables are in excel file**

**Table 1:** Analytical characteristics of the 205 triacylglycerides identified in human milk and example reference if previously published

**Table 2:** Human milk triacylglycerides with significant concentration changes throughout the morning feed

**Table 3:** Human milk triacylglycerides with significant concentration changes throughout the evening feed

**Table 4:** Average concentrations of all 205 triacylglycerides identified in each sample type

**Table 5:** Average infant daily dose for the 205 triacylglycerides identified

**
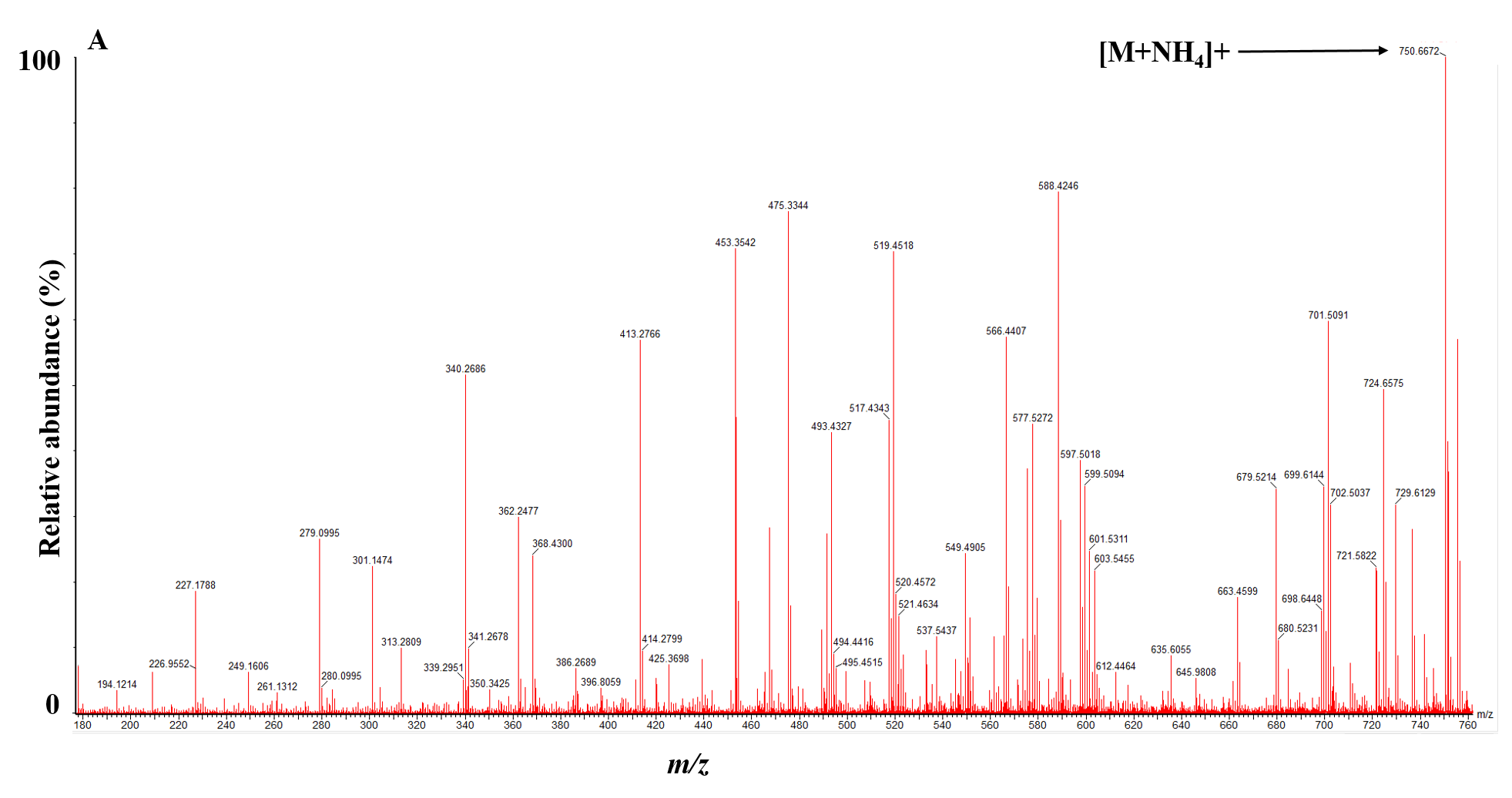
Supplementary Figure 1.** A) Mass spectra for TAG(12:0_/**17:1**/_14:1) (TAG192) from *m/z* 180 to 760 at 15.3 minutes, with low collision energy (20eV). Maximum (100%) abundance is 2.34e4. Ammonium adduct precursor ion is present, *m/z* 750.6672. Spectra contains many overlapping ions.

**
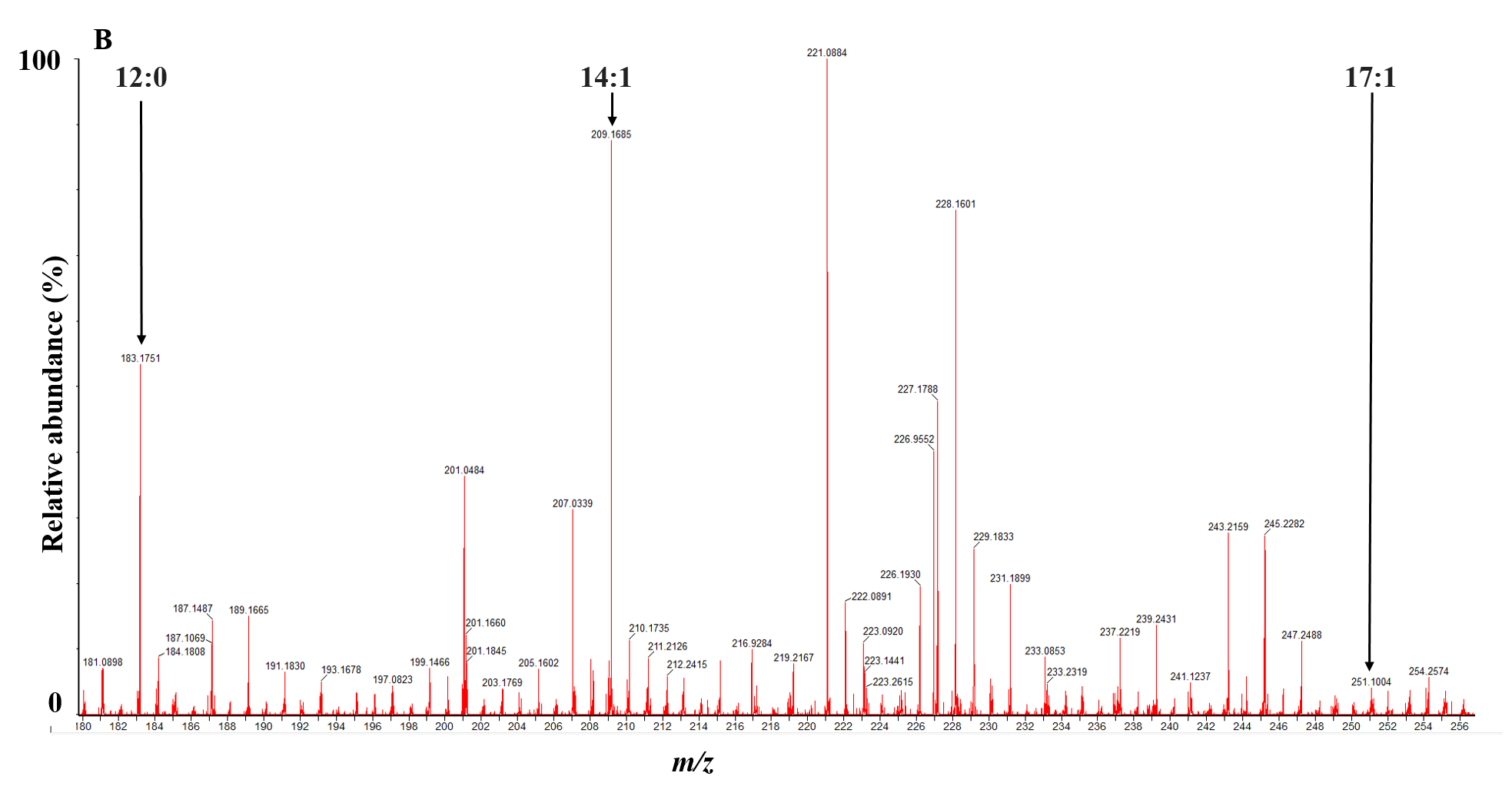
**

**Supplementary Figure 1.** B) Corresponding mass spectra for TAG(12:0_/**17:1**/_14:1) (TAG192) with high collision energy (55eV), from *m/z* 180 to 256. Maximum (100%) abundance is 4.17e3. When fragment ions for this TAG are searched, the most abundant is *m/z* 209.1685 [CH_3_(CH_2_)_4_CH=CH(CH_2_)_6_C=O]^+^ for FA 14:1; the second most abundant is *m/z* 183.1751 [CH_3_(CH_2_)_10_C=O]^+^ for FA 12:0; the least abundant is *m/z* 251.1004 [CH_3_(CH_2_)_6_CH=CH(CH_2_)_7_C=O]^+^ for FA 17:1. This tentatively identifies 17:1 as the FA in the Sn-2 position.
